# Supplementary figures and images for: A digital twin reproducing gene regulatory network dynamics of early Ciona embryos indicates robust buffers in the network
Source: PLoS Genet. 2023 Sep 27;19(9):e1010953. doi: 10.1371/journal.pgen.1010953 (PMC10530022; doi:10.1371/journal.pgen.1010953)

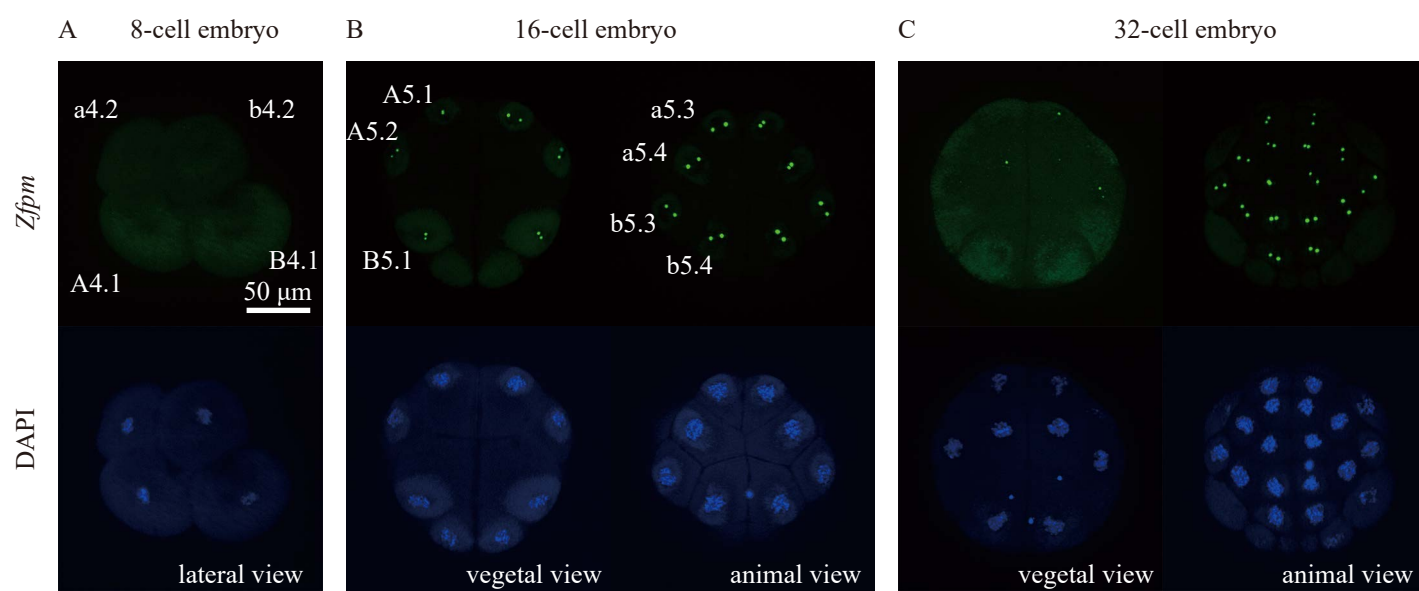

Supplement: S1 Fig — Fluorescence in situ hybridization was used to examine expression patterns of Zfpm in (A) 8-cell, (B) 16-cell, and (C) 32-cell embryos. Note that expression is detected as green dots in nuclei of cells in the animal and vegetal hemispheres of a 16-cell embryo and in the animal hemisphere of a 32-cell embryo, but not in the 8-cell embryo or in the vegetal hemisphere of the 32-cell embryo. Nuclei are stained with DAPI (blue). (PDF) [file pgen.1010953.s001.pdf]

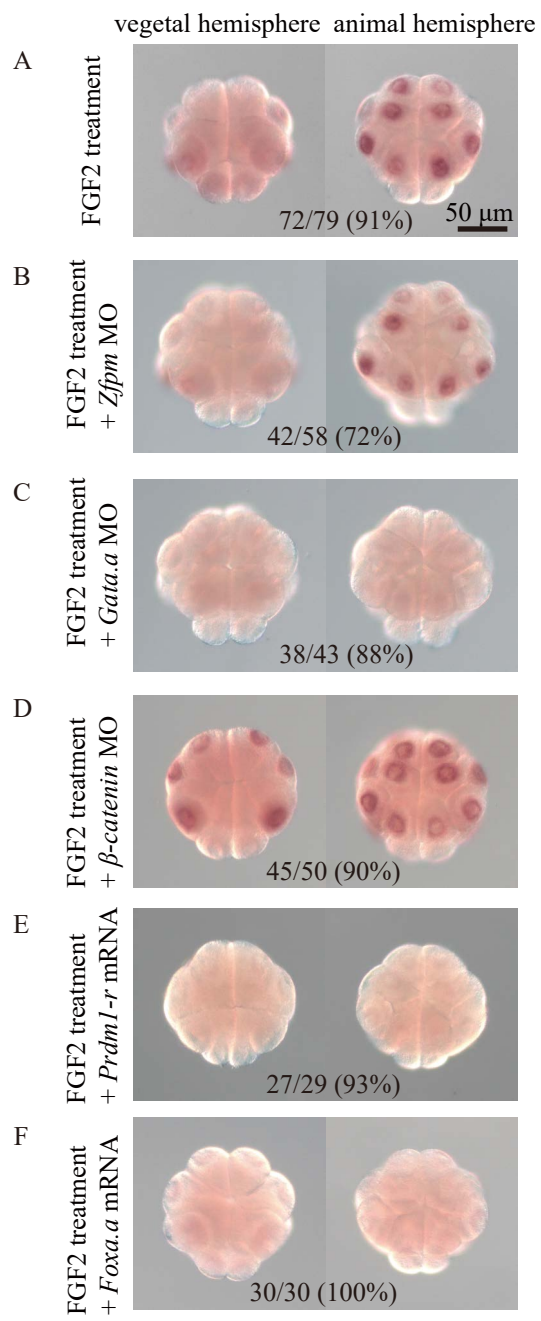

Supplement: S4 Fig — Nodal expression was examined with in situ hybridization at the 16-cell stage in (A) an embryo treated with FGF2, (B) an embryo injected with the Zfpm MO and treated with FGF2, (C) an embryo injected with the Gata.a MO and treated with FGF2, (D) an embryo injected with the β-catenin MO and treated with FGF2, (E) an embryo injected with Prdm1-r mRNA and treated with FGF2, (F) an embryo injected with Foxa.a mRNA and treated with FGF2. Nodal expression in unperturbed control embryos is shown in Fig 3D. Photographs in (A) are the same as those in Fig 2C. Total numbers of embryos examined and numbers of embryos that photographs represent are shown within the panels. (PDF) [file pgen.1010953.s004.pdf]

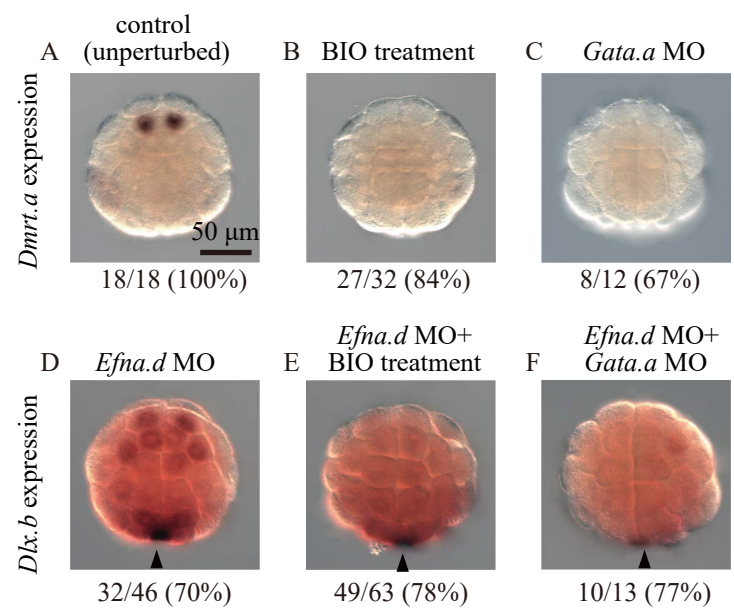

Supplement: S6 Fig — (A) Dmrt.a expression in normal unperturbed embryos. (B) Dmrt.a expression is abolished in embryos incubated in sea water containing BIO, an inhibitor of Gsk3; therefore, β-catenin is expected to be stabilized in all cells. (C) Dmrt.a expression is also abolished in embryos injected with the Gata.a MO. (D) Dlx.b expression is detected in anterior cells of the animal hemisphere of embryos injected with a MO against Efna.d, as we reported before [5]. (E) Dlx.b expression is not detected in embryos injected with the Efna.d MO and incubated in sea water containing BIO. (F) Dlx.b expression was not detected in embryos injected with the Efna.d MO and the Gata.a MO. Arrowheads indicate maternal Dlx.b mRNA localized to the posterior pole. Total numbers of embryos examined and numbers of embryos that photographs represent are shown below. (PDF) [file pgen.1010953.s006.pdf]
