# Supplementary material for: A digital twin reproducing gene regulatory network dynamics of early Ciona embryos indicates robust buffers in the network
Source: PLoS Genet. 2023 Sep 27;19(9):e1010953. doi: 10.1371/journal.pgen.1010953 (PMC10530022; doi:10.1371/journal.pgen.1010953)

A

Stage	Batch	% mutations (TIDE analysis)	3/6/9 base-indels (TIDE analysis)	% effective and homozygous mutations	Embryos that lost *Nodal* expression
Larva	1	69.6%	Not further analyzed		
	2	74.4%	Not further analyzed		
32-cell embryo	3	69.5%	11.2%	34.0%	20.7% (6/29)
	4	52.9%	12.9%	16.0%	18.9% (10/53)
	5	45.6%	6.1%	15.6%	10 .0 % (3/30)
	6	12.9%	Not further analyzed		
	7	1.1%	Not further analyzed		
	8	21.6%	Not further analyzed		
	9	16.2%	Not further analyzed		
	10	33.1%	Not further analyzed		
	11	14.6%	Not further analyzed		

B

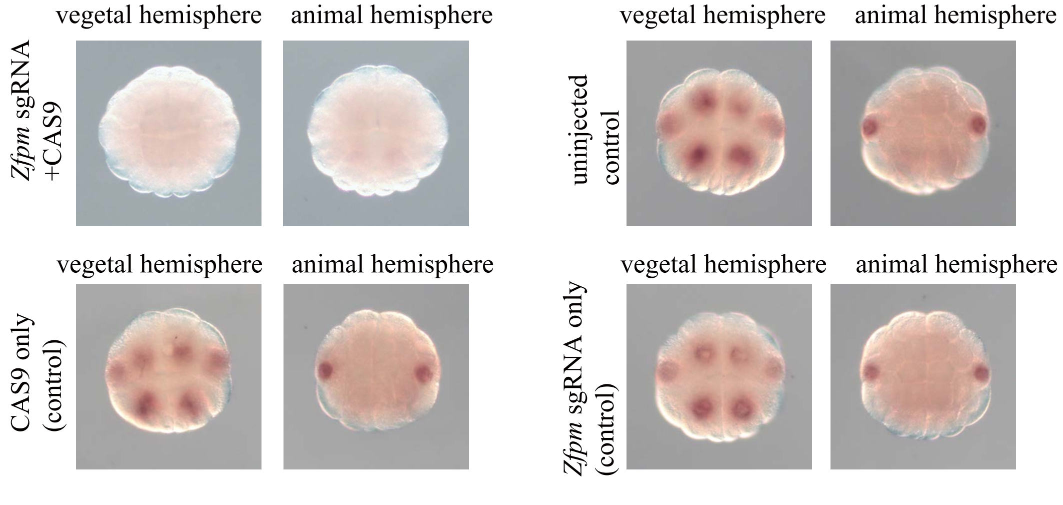

Supplement: S2 Fig — (A) First, we examined genomic DNA of larvae developed from eggs injected with Cas9 protein and a guide RNA designed to bind to the region encoding the fifth and sixth zinc fingers of Zfpm. Sequencing followed by TIDE analysis [45] indicated that 69.6 and 74.4% of DNA fragments amplified with PCR from two batches of larvae contained mutations, suggesting that this guide RNA was effective. Next, we performed the same experiments using 16-cell embryos. Mutagenic efficiency varied from 1.1 to 69.5% among nine batches of embryos we examined. Therefore, we used three batches of embryos that showed high mutagenic efficiencies (69.5, 52.9, and 45.6%) for the following analyses. TIDE further indicated that these three batches contained 3/6/9 base insertions or deletions in 11.2%, 12.9%, and 6.1% of the amplified DNA fragments. Because these mutations did not cause frame-shifts, and may not have severely impaired Zfpm function, we conservatively estimated that 58.3 (= 69.5–11.2), 40.0 (= 52.9–12.9), and 39.5 (= 45.6–6.1) % of these embryos contained effective mutations. As all cells are diploid, 34.0, 16.0, and 15.6% of cells were estimated to contain effective mutations in both maternal and paternal alleles. By in situ hybridization, we found that 20.7, 18.9, and 10.0% of embryos in these batches lost Nodal expression. These percentages were close to the expected percentages, indicating that Zfpm is required for Nodal expression and that the Zfpm MO acted specifically. (B) An embryo that lost Nodal expression by CRISPR knockout of Zfpm. Expression was examined with in situ hybridization. An uninjected embryo (n = 43) and embryos injected with either Cas9 (n = 45) or Zfpm sgRNA (n = 39) are shown as controls. Nodal expression was not changed in these controls. (PDF) [file pgen.1010953.s002.pdf]
