# Supplementary material for: A digital twin reproducing gene regulatory network dynamics of early Ciona embryos indicates robust buffers in the network
Source: PLoS Genet. 2023 Sep 27;19(9):e1010953. doi: 10.1371/journal.pgen.1010953 (PMC10530022; doi:10.1371/journal.pgen.1010953)
Supplement: S3 Fig — This tentative function was determined previously [5], and explains the Nodal expression pattern in normal conditions at the 16-cell and 32-cell stages and in a variety of experimental conditions at the 32-cell stage, but cannot accurately predict expression patterns in experimental conditions at the 16-cell stage. Note that expression patterns of Prdm1-r and Foxa.a indicated that either of them or both are involved in the fourth conjunctive clause, but their involvement was not strictly tested [5]. (PDF) [file pgen.1010953.s003.pdf]

Foxa.a∧Fgf9/16/20∧β-catenin ∨	⟶	A6.1/A6.3/B6.1
Tbx6-r.b∧β-catenin ∨	⟶	B6.1
Sox1/2/3∧¬Foxa.a∧¬Foxd∧Fgf9/16/20∧¬Efna.d∧¬β-catenin∧Gata.a ∨	⟶	b6.5
Fgf9/16/20∧¬Gdf1/3-r∧¬Admp∧¬Prdm1-r/¬Foxa.a	⟶	Expression in experimental conditions
