# Supplementary material for: A digital twin reproducing gene regulatory network dynamics of early Ciona embryos indicates robust buffers in the network
Source: PLoS Genet. 2023 Sep 27;19(9):e1010953. doi: 10.1371/journal.pgen.1010953 (PMC10530022; doi:10.1371/journal.pgen.1010953)
Supplement: S5 Fig — (A) RFs for Lhx3/4, Neurogenin, and Dickkopf are commonly represented as Foxd⋀Fgf9/16/20⋀β-catenin, and these genes are expressed in A6.1, A6.3, and B6.1 of normal embryos. Regulatory functions represented as Fgf9/16/20⋀β-catenin and Foxd⋀β-catenin can induce the same expression patterns. (B-D) In cases in which (B) Foxd, (C) Fgf9/16/20, or (D) β-catenin acts in all cells of 16-and 32-cell embryos, three RFs induce different expression patterns. This observation indicates that Lhx3/4, Neurogenin, and Dickkopf could be expressed differently upon changes of distribution patterns of their upstream factor through rewiring that does not change expression patterns in normal embryos. (PDF) [file pgen.1010953.s005.pdf]

A

	Cells	Foxd	Fgf9/16/20	β-catenin	Foxd∧Fgf9/16/20∧β-catenin	Fgf9/16/20∧β-catenin	Foxd∧β-catenin
16-cell embryo	A5.1, A5.2	0	0	1	0	0	0
	B5.1	0	0	1	0	0	0
	a5.3, a5.4, b5.3, b5.4	0	0	0	0	0	0
32-cell embryo	A6.1, A6.3	1	1	1	1	1	1
	A6.2, A6.4	1	1	0	0	0	0
	a6.5	0	1	0	0	0	0
	a6.6–a6.8	0	1	0	0	0	0
	B6.1	1	1	1	1	1	1
	B6.2	1	1	0	0	0	0
	B6.4	0	1	0	0	0	0
	b6.5	0	1	0	0	0	0
	b6.6–b6.8	0	1	0	0	0	0

B

	Cells	Foxd	Fgf9/16/20	β-catenin	Foxd∧Fgf9/16/20∧β-catenin	Fgf9/16/20∧β-catenin	Foxd∧β-catenin
16-cell embryo	A5.1, A5.2	1	0	1	0	0	1
	B5.1	1	0	1	0	0	1
	a5.3, a5.4, b5.3, b5.4	1	0	0	0	0	0
32-cell embryo	A6.1, A6.3	1	1	1	1	1	1
	A6.2, A6.4	1	1	0	0	0	0
	a6.5	1	1	0	0	0	0
	a6.6–a6.8	1	1	0	0	0	0
	B6.1	1	1	1	1	1	1
	B6.2	1	1	0	0	0	0
	B6.4	1	1	0	0	0	0
	b6.5	1	1	0	0	0	0
	b6.6–b6.8	1	1	0	0	0	0

C

	Cells	Foxd	Fgf9/16/20	β-catenin	Foxd∧Fgf9/16/20∧β-catenin	Fgf9/16/20∧β-catenin	Foxd∧β-catenin
16-cell embryo	A5.1, A5.2	0	1	1	0	1	0
	B5.1	0	1	1	0	1	0
	a5.3, a5.4, b5.3, b5.4	0	1	0	0	0	0
32-cell embryo	A6.1, A6.3	1	1	1	1	1	1
	A6.2, A6.4	1	1	0	0	0	0
	a6.5	0	1	0	0	0	0
	a6.6–a6.8	0	1	0	0	0	0
	B6.1	1	1	1	1	1	1
	B6.2	1	1	0	0	0	0
	B6.4	0	1	0	0	0	0
	b6.5	0	1	0	0	0	0
	b6.6–b6.8	0	1	0	0	0	0

D

	Cells	Foxd	Fgf9/16/20	β-catenin	Foxd∧Fgf9/16/20∧β-catenin	Fgf9/16/20∧β-catenin	Foxd∧β-catenin
16-cell embryo	A5.1, A5.2	0	0	1	0	0	0
	B5.1	0	0	1	0	0	0
	a5.3, a5.4, b5.3, b5.4	0	0	1	0	0	0
32-cell embryo	A6.1, A6.3	1	1	1	1	1	1
	A6.2, A6.4	1	1	1	1	1	1
	a6.5	0	1	1	0	1	0
	a6.6–a6.8	0	1	1	0	1	0
	B6.1	1	1	1	1	1	1
	B6.2	1	1	1	1	1	1
	B6.4	0	1	1	0	1	0
	b6.5	0	1	1	0	1	0
	b6.6–b6.8	0	1	1	0	1	0
