# Supplementary material for: A digital twin reproducing gene regulatory network dynamics of early Ciona embryos indicates robust buffers in the network
Source: PLoS Genet. 2023 Sep 27;19(9):e1010953. doi: 10.1371/journal.pgen.1010953 (PMC10530022; doi:10.1371/journal.pgen.1010953)
Supplement: Attachment — Submitted filename: Response.pdf [file pgen.1010953.s009.pdf]

[Editors' comment]

The manuscript was fully evaluated at the editorial level and by three independent peer reviewers. We apologize for the length of this process. The reviewers appreciated your work. Referee 2 supports its publication, but referees 1 and 3 raised substantial concerns about the current manuscript. Referee 3, in particular, is worried that the way you constructed the networks in this and in your previous study (Tokuoka et al., 2021) may artificially favour the selection of redundant networks, which would undermine a major message of your study. Referee 1, besides encouraging you to revise/precise many sentences, is worried that the use of boolean networks may provide little more insight than providing a mere restatement of experimental results.

Based on these significant concerns, we will not be able to accept this version of the manuscript, but we would be willing to review a much-revised version that in particular addresses these two major concerns. We cannot, of course, promise publication at that time.

[Response]

As to the first point raised by reviewer 3: This reviewer has a concern that the network we reproduced might favor redundancy artificially. We do not think that this is the case with the following reasons. First, the upstream factors we used were chosen by an unbiased way. Namely, we analyzed regulatory factors that are (or are expected to be) expressed at the 32-cell stage. We did not intentionally pick or exclude specific regulatory factors. Second, our exhaustive search identified the simplest Boolean function in the DNF among all possible combinations. Hence, this method cannot be biased to pick redundancy. Third, all theoretically redundant factors are indeed involved in regulation of target genes. For example, *Lhx3/4* is regulated by Foxd, Fgf9/16/20, and β-catenin (thereby the *Lhx3/4* RF is represented by Foxd&Fgf9/16/20&β-catenin), and *Lhx3/4* expression is in fact abolished in embryos injected with a MO against Foxd or Fgf9/16/20. Meanwhile, we found that Foxd&β-catenin or Fgf9/16/20&β-catenin theoretically gave the same expression pattern as Foxd & Fgf9/16/20 & β-catenin. This means that Foxd and Fgf9/16/20 give theoretically redundant information. Namely, while these two factors give theoretically redundant information, these two factors are necessary for expression of *Lhx3/4* in real embryos. We do not think that this process is biased to favor redundancy. Please understand that we say that these factors give theoretically redundant information to target genes and we never say that these factors

are dispensable in real embryos. Fourth, from these identified theoretically redundant factors, we picked an example, Zfpm for *Nodal* expression, for substantiating the hypothesis; if the regulatory region of *Nodal* is properly re-designed, it directs expression in the same cells without Zfpm. Indeed, the upstream region of *Nodal* directed expression in b6.5, even after it was changed to be independent of Zfpm. This example indicates that the information given by Zfpm is indeed redundant.

We explained these points in detail in the revised version of the manuscript (Line 272–288). We believe that the concern raised by Reviewer 3 is now resolved.

As to the second point raised by Reviewer 1: The Boolean regulatory function we determined is NOT "a mere restatement of experimental results". First, we considered 16 upstream factors in our previous study, and 18 factors in the present study (Zfpm and Gata.a are considered additionally). This means that there are 65536 (2^{16}) or 262144 (2^{18}) combinations of activities of upstream factors in the binary space. It is unrealistic to test all these combinations in experiments using real embryos, and we indeed determined the regulatory functions using data from a limited number of experiments. That is, the regulatory functions predict gene expression in many unexamined conditions. This predictive power means that these are not mere restatements of experimental results. For example, *Lhx3/4* function is written as Foxd & Fgf9/16/20 & β-catenin. This indicates not only that these three factors are involved in *Lhx3/4* expression, but also that the remaining upstream factor candidates are not necessary for *Lhx3/4* expression. Second, we created the gene expression pattern of b6.5, which is a cell of 32-cell embryos, in the animal hemisphere of 16-cell embryos, according to the calculation using the regulatory functions. We did not use 16-cell embryos for experiments to determine the regulatory functions. We did not perform the same experiments even at the 32-cell stage, except for *Hes.b*. Therefore, this is not a repeat of what we have done in experiments to determine the regulatory functions. We think that such reproduction cannot be achieved without the regulatory functions. Third, the regulatory functions enabled us to find that temporal or spatial information are redundantly given to target genes in many cases. This analysis, which simulated rewiring of the network, is impossible without the regulatory functions. With these reasons, we do not agree with the opinion that the regulatory functions are mere restatements of experimental results. We added a brief statement of this point as follows:

(Line 343–345) Thus, the RFs predict gene expression patterns under untested conditions; therefore, the RFs are not mere restatements of experimental results.

Reviewer #1

[Reviewer's comment]

I have now carefully read this manuscript. My major objection is that the English is, at times, rather hard to read. The article provides an improvement on a previous related article. The improvement consist in the inclusion of one gene, Nodal, and an exploration of the redundancy in the regulation of the patterns of gene expression in the 32 cell stage. There are really not major take-home message rather than a slightly more detailed description of the underlying network (e.g. no actual cell signaling occurring). I am a theoretical biologist and I do not see much benefit in the boolean model. In practice it is just a re-statement of experimental findings. This is certainly not useless and the virtual twin can be helpful as a kind of summary for Ciona researchers. I am uncertain as to whether this manuscript provides enough incremental insight as to deserve publication in PLOS Genetics, I leave that decision to reviewers that work experimentally with Ciona.

I have a number of minor comments. In truth, these are only the major comments among the minor ones because there are quite many grammar mistakes and obscure sentences over the article. The article needs to revised by a native English speaker.

[Response]

We apologize for mistakes. We again asked a native English speaker with a PhD to proofread the revised version of the manuscript.

Meanwhile, we do not agree with the opinion that our models are "a re-statement of experimental findings", as we wrote in our response to Editor's comment, with the following reasons.

First, we considered 16 upstream factors in our previous study, and 18 factors in the present study (Zfpm and Gata.a are considered additionally). This means that there are 65536 (2^{16}) or 262144 (2^{18}) combinations of activities of upstream factors in the binary space. It is unrealistic to test all these combinations in experiments using real embryos, and we indeed determined the regulatory functions using data from a limited number of experiments. That is, the regulatory functions predict gene expression in many

unexamined conditions. This predictive power means that these are not mere restatements of experimental results. For example, *Lhx3/4* function is written as Foxd & Fgf9/16/20 & β-catenin. This indicates not only that these three factors are involved in *Lhx3/4* expression, but also that the remaining upstream factor candidates are not necessary for *Lhx3/4* expression.

Second, we created the gene expression pattern of b6.5, which is a cell of 32-cell embryos, in the animal hemisphere of 16-cell embryos, according to the calculation using the regulatory functions. We did not use 16-cell embryos for experiments to determine the regulatory functions. We did not perform the same experiments even at the 32-cell stage, except for *Hes.b*. Therefore, this is not a repeat of what we have done in experiments to determine the regulatory functions. We think that such reproduction cannot be achieved without the regulatory functions.

Third, the regulatory functions enabled us to find that temporal or spatial information are redundantly given to target genes in many cases. This analysis, which simulated rewiring of the network, is impossible without the regulatory functions.

[Reviewer's comment]

Minor:

-"Although the structure of such networks, which represent connections among regulatory genes, has been studied extensively, it is unclear how these networks encode gene expression dynamics.". It is unclear what "encode" is supposed to mean in this sentence, I think encode is not the right word in this context.

[Response]

We changed the sentence as follows:

(Original) Although the structure of such networks, which represent connections among regulatory genes, has been studied extensively, it is not fully understood how these networks encode gene expression dynamics.

(Revised) Although the structure of such networks, which represent connections among regulatory genes, has been studied extensively, it is not fully understood how these networks **control** gene expression dynamics. (Line 44–46)

[Reviewer's comment]

-In line 48, It is just incorrect to label a phylogenetic group as "basal". Please correct and check: https://pubmed.ncbi.nlm.nih.gov/16733736/

[Response]

We removed 'basal' as follows:

(Original) Ascidians are ideal **basal** chordates for studying gene regulatory networks because of their simple genome structure and simple embryonic structure.

(Revised) Ascidians are ideal chordates for studying gene regulatory networks because of their simple genome structure and simple embryonic structure. (Line 48–49)

[Reviewer's comment]

-I think the reading of the article would be facilitated by a better explanation or more detailed discussion of this statement: "Regulatory mechanisms for 12 of these 13 genes have been described mathematically as Boolean functions, 56 which represent necessary and sufficient conditions for their expression (Tokuoka et al., 2021) (see an example for Boolean functions in Figure 2A)."

[Response]

According to the advice, we added the following sentence:

(Line 59–60) Expression patterns of these 12 genes can be predicted by calculations using these functions and activity of upstream factors.

[Reviewer's comment]

-This sentence is not clear enough: "The regulatory function (RF) for the remaining gene, Nodal, was also tentatively determined; it represents necessary conditions, but does not represent sufficient conditions (Tokuoka et al., 2021). ". What is not necessary or sufficient? And for what?

[Response]

To clarify the point, we revised this part as follows:

> (Original) The regulatory function (RF) for the remaining gene, *Nodal*, was also tentatively determined; it represents necessary conditions, but does not represent sufficient conditions (Tokuoka et al., 2021)

> (Revised) The RF for the remaining gene, *Nodal*, has also been determined provisionally. Necessary factors for *Nodal* expression were identified by our exhaustive examination of functions of regulatory genes expressed before the 32-cell stage. However, we did not always succeed in inducing *Nodal* expression in real embryos in conditions in which the tentative RF predicted *Nodal* activation (Tokuoka et al., 2021). This suggests that an additional modulator for transcription factors or signaling pathways may be involved in *Nodal* regulation. (Line 60–66)

[Reviewer's comment]

-This sentence is also unclear: "In the present study, we first devised such a digital twin and then showed that gene expression patterns can indeed be manipulated according to predictions from the digital twin." Are the authors meaning that they are going to perform experimental manipulations? This is clearer later but it should be clear already at this point.

[Response]

According to the advice, we revised this part as follows:

(Original) In the present study, we first devised such a digital twin and then showed that gene expression patterns can indeed be manipulated according to predictions from the digital twin.

(Revised) In the present study, we first devised such a simulator and then showed that gene expression patterns can indeed be manipulated **in real embryos according to predictions from the simulator.** (Line 69–71)

[Reviewer’s comment]

-The following sentence is also unclear: “We found robust buffering of regulatory mechanisms, which potentially permits rewiring of GRNs without changing expression patterns.” What is a regulatory mechanism for the authors? How it relates to Rfs. What do they mean by “buffering”, this word is written as a verb form. This suggests that buffering is an active thing in this case but this sound a bit confusing and it is anyway unclear what, if anything, is doing the buffering.

[Response]

To clarify what we mean by “regulatory mechanisms” and “RFs”, we revised the following part:

(Original) Regulatory mechanisms for 12 of these 13 genes have been described mathematically as Boolean functions, which represent necessary and sufficient conditions for their expression (Tokuoka et al., 2021) (see an example for Boolean functions in Figure 2A).

(Revised) Regulatory mechanisms for 12 of these 13 genes have been described mathematically as Boolean functions, which represent necessary and sufficient conditions for their expression (Tokuoka et al., 2021) (see an example for Boolean functions in Figure 2A). **In other words, combinations of upstream regulatory factors that induce expression of these genes are represented as Boolean formulae, which we call regulatory functions (RFs).** (Line 54–59)

We replaced “buffering” with “buffers” according to the advice.

[Reviewer’s comment]

-It is not totally clear what the authors mean by a gene’s RF. The authors provide no explicit definition.

[Response]

We call Boolean formulae that represent regulatory mechanisms for activating genes in specific cells at specific time as regulatory functions (RFs). Please see our answer to the preceding criticism.

[Reviewer’s comment]

-I cannot understand this statement: “The finding that RFs are successfully represented as Boolean functions indicates that qualitative, but not quantitative, control are important for regulation in early ascidian embryos (Tokuoka et al., 2021).” The last part after “control” is objectively not-understandable. This is probably because the authors use control and regulation in non-standard ways, that need to be clarified.

[Response]

We revised the sentence as follows:

(Original) The finding that RFs are successfully represented as Boolean functions indicates that qualitative, but not quantitative, control are important for regulation in early ascidian embryos (Tokuoka et al., 2021).

(Revised) The finding that RFs are successfully represented as Boolean functions indicates that qualitative, but not quantitative, control is important for **gene expression** in early ascidian embryos (Tokuoka et al., 2021). (Line 98–100)

[Reviewer's comment]

-The grammar in this statement is improvable: "We assumed that cells descended from cells expressing a transcription factor gene at the 16-cell stage express the encoded protein at the 32-cell stage because of a delay between gene expression and protein translation (Tokuoka et al., 2021)."

[Response]

We revised the sentence as follows:

(Original) We assumed that cells descended from cells expressing a transcription factor gene at the 16-cell stage express the encoded protein at the 32-cell stage because of a delay between gene expression and protein translation (Tokuoka et al., 2021).

(Revised) We assumed that **descendants of** cells expressing a transcription factor gene at the 16-cell stage express the encoded protein at the 32-cell stage because of a delay between gene expression and protein translation (Tokuoka et al., 2021). (Line 106–108)

[Reviewer's comment]

-Is "artifactitiously" an actual word? How about just "artificially"

[Response]

We removed this word (Line 152).

[Reviewer's comment]

-The one-paragraph sentence between lines 154 and 164 needs to be broken into several different sentences, otherwise it is completely unredeable.

[Response]

We revised this part as follows:

(Original) Using a method that we previously developed (Tokuoka et al., 2021), the distribution pattern of upstream factors inferred from observations in previous studies (Hudson et al., 2013; Imai et al., 2016; Kumano et al., 2011; Oda-Ishii et al., 2018; Oda-Ishii et al., 2016; Oda-Ishii and Satou, 2018; Ohta and Satou, 2013; Ohta et al., 2015; Rothbächer et al., 2007; Shirae-Kurabayashi et al., 2011; Tassy et al., 2006; Tokuoka et al., 2018; Yagi et al., 2004), an assumption that cells descended from cells expressing a transcription factor gene at the 16-cell stage would express the encoded protein at the 32-cell stage (S1 Table), and experimental results for *Nodal* expression in various conditions, which we described above and in our previous study (Tokuoka et al., 2021) (S1 Table), we determined the simplest DNF of the Boolean function for *Nodal* (Figure 2A).

(Revised) Using a method that we previously developed (Tokuoka et al., 2021) and experimental results for *Nodal* expression in various conditions, which we described above and in our previous study (Tokuoka et al., 2021) (S1 Table), we determined the simplest DNF of the Boolean function for *Nodal* (Figure 2A). For this calculation, we assumed that descendants of cells expressing a transcription factor gene at the 16-cell stage would express the encoded protein at the 32-cell stage (S1 Table), and used the distribution pattern of upstream factors inferred from observations in previous studies (Hudson et al., 2013; Imai et al., 2016; Kumano et al., 2011; Oda-Ishii et al., 2018; Oda-Ishii et al., 2016; Oda-Ishii and Satou, 2018; Ohta and Satou, 2013; Ohta et al., 2015; Rothbächer et al., 2007; Shirae-Kurabayashi et al., 2011; Tassy et al., 2006; Tokuoka et al., 2018; Yagi et al., 2004). (Line 172–181)

[Reviewer’s comment]

-The title of this section “Reproduction of the pattern of a cell of a 32-cell-embryo in 16-cell embryos” is written in a rather strange manner.

[Response]

We revised the section title as follows:

(Original) Reproduction of the pattern of a cell of a 32-cell-embryo in 16-cell embryos

(Revised) Reproduction of the pattern of a cell **with neural fate** of a 32-cell embryo **at the 16-cell stage** (Line 205)

[Reviewer's comment]

-In line 231: "Because RFs are encoded in cis-regulatory regions,…" Is that the case? Rfs can also be regulated by protein-protein interactions occurring between transcriptional factors and co-factors, as the authors results seem to indicate for the regulation of Nodal.

[Response]

According to the advice, we added "primarily" to the sentence. Contrary to the criticism, in the case of *Nodal*, Zfpm-dependency is encoded in cis-regulatory region. Zfpm is a co-factor of Gata.a, but as we showed Gata.a sites in the upstream region of *Nodal* is Zfpm-dependent, and Gata.a sites in the upstream region of *Zic-r.b* is Zfpm-independent, which means that cis-regulatory sequences determine which Gata.a sites are dependent on or independent of Zfpm..

(Original) Because RFs are encoded in *cis*-regulatory regions, often in a complex manner and redundantly (Hong et al., 2008; Oda-Ishii et al., 2005; Oudelaar and Higgs, 2021; Wittkopp and Kalay, 2012; Yuh et al., 2001), it is generally difficult to change regulatory mechanisms or RFs *in vivo*.

(Revised) Because RFs are **primarily** encoded in *cis*-regulatory regions, often in a complex and redundant manner (Hong et al., 2008; Oda-Ishii et al., 2005; Oudelaar and Higgs, 2021; Wittkopp and Kalay, 2012; Yuh et al., 2001), it is generally difficult to change regulatory mechanisms or RFs *in vivo*. (Line 245–248)

[Reviewer's comment]

-"For RFs for the 13 genes, 17 regulators appear 99 times in total." This sentence is not understandable.

[Response]

We revised the sentence as follows:

(Original) For RFs for the 13 genes, 17 regulators appear 99 times in total.

(Revised) In RFs for the 13 genes, 17 **upstream** regulators appear 99 times in total. **Each of these 17 regulators appears multiple times in different conjunctions (Figure 4).** (Line 250–251)

[Reviewer's comment]

-In line 244: "This finding suggests a possibility that the observed redundancy provides 245a basis for developmental system drifts." This is statement is true but the reverse can also be true: this redundancy may be the result of developmental systems drift, not just a possible cause.

[Response]

We agree with the alternative possibility. However, we think that this alternative possibility is more unlikely. This possibility assumes that three independent changes in regulatory regions of *Lhx3/4*, *Neurogenin*, and *Dickkopf* would have resulted in the same regulatory function for example. To explain this, we added the following sentences:

(Line 265–269) Conversely, it is theoretically possible that developmental system drift created this redundancy. However, this latter possibility is unlikely at least in cases of *Lhx3/4*, *Neurogenin*, and *Dickkopf*. If so, it would indicate that developmental system drift changed regulatory mechanisms of these genes independently three times, so that these three genes became controlled by the same combination of regulatory factors.

[Reviewer's comment]

-In line 246: "The digital twin also indicated that such rewiring enabled these genes to behave differently from one another if expression patterns of upstream factors were changed (S5B-D Fig)" The verb tenses seem to be off in this sentence. From the writing is seems that that is what happened in nature in the past, while that happened only in the model.

[Response]

According to the reviewer's advice, we revised the following sentence:

(Original) The digital twin also indicated that such rewiring enabled these genes to behave differently from one another if expression patterns of upstream factors were changed (S5B-D Fig)

(Revised) The simulator also indicated that such rewiring **would** enable these genes to behave differently if expression patterns of upstream factors were changed (S5B-D Fig). (Line 263–265)

[Reviewer's comment]

-In line 248: "Although combinations of regulators may not be the only evolutionary constraints of Rfs," It is unclear what the authors mean by "combinations of regulators" and "evolutionary constraints" in the context of this sentence.

[Response]

We removed these phrases from the sentence.

(Original) **Although combinations of regulators may not be the only evolutionary constraints of RFs,** the results in the present study indicated that more than half the regulators give theoretically redundant temporal or spatial information to target genes, or perhaps that some do not furnish such information.

(Revised) Thus, the above results indicate that more than half the regulators give theoretically redundant temporal or spatial information to target genes, or that some do not furnish such information. (Line 269–271)

[Reviewer’s comment]

-I wonder to which extend the robustness results are a mere consequence of not getting the RF right for all genes. Ideally, the reader would not have to read all previous articles by the authors to figure out whether this is the case or not.

[Response]

The present and previous studies have shown that all of these redundant factors indeed act to regulate the 13 genes by experiments using real embryos. Therefore, we think that these redundant factors we found really give redundant information. To clarify this point, we added the following sentences:

> (Line 279–285) The above computational analysis showed that Foxd and Fgf9/16/20 theoretically gave redundant cues for regulation of *Lhx3/4*, whereas *Lhx3* expression is lost in embryos injected with an MO against *Foxd* or *Fgf9/16/20* (Tokuoka et al., 2021). Similarly, the present and previous studies have experimentally shown that each of the observed redundant factors act to establish specific expression patterns of the 13 target genes (Bertrand et al., 2003; Imai et al., 2016; Ohta and Satou, 2013; Ohta et al., 2015; Tokuhiro and Satou, 2021; Tokuhiro et al., 2017; Tokuoka et al., 2021).

Reviewer #2

[Reviewer's comment]

In this manuscript, Tokuoka and Satou present an updated version of their 16-32-cell stage digital twin embryo. Previously, they unraveled the simplest combinations of "regulatory functions" (RF) for 12 out of 13 regulatory genes/zones, activated at the 32-cell stage, using Boolean logic (Tokuoka et al, 2021). Here, they extend this analysis to cover the 13th gene, Nodal, completing its RF, with the addition of the regulator Zfpm (fog). The authors convincingly tested their predicted Nodal RF (combination of regulatory factors/conditions sufficient for Nodal expression) using the digital twin embryo and real embryo experiments. Next, the authors modified the theoretical RFs for all 13 genes and found that in 55 cases in which a single regulator was removed, the digital twin still predicted the correct gene expression output. In other words, in terms of computation, many factors had 'redundant' coding functions. The authors propose the hypothesis that this type of redundant coding function could be a mechanism that would allow developmental systems drift, since changes in regulatory sequences, when coupled with cis-regulatory changes, should still allow the same phenotypic output. Furthermore, the authors then test in embryos the hypothesis that removal of redundant factors will lead to the same phenotypic output if the cis-regulatory sequences are also adapted. They did this by swapping the Gata.a binding sites in the Nodal b6.5 enhancer (dependence on Zfpm) with those of Zic-r.b (independence on Zfpm), which confirmed their hypothesis. The updated HTML-based program is improved and easy to use and will be very useful for the community.

The manuscript is carefully conducted and interpretation is clever and interesting. I think that this thought-provoking manuscript will be of great interest for both ascidian developmental biologists and the broad audience of evo-devo researchers.

[Response]

We thank this reviewer for his/her strong support.

[Reviewer's comment]

Minor comments.

Line 23: Sentence is not clear, I would replace 'or developmental system drift' with 'enabling (or allowing) developmental systems drift'.

[Response]

Following the advice, we changed the sentence as follows:

(original) Such robust buffering of regulatory mechanisms may provide a basis of rewiring of GRNs without changing expression patterns of downstream genes, or developmental system drift, during evolution.

(revised) Such robust buffers of regulatory mechanisms may provide a basis of **enabling** developmental system drift, or rewiring of GRNs without changing expression patterns of downstream genes, during evolution. (Line 23–25)

[Reviewer's comment]

line 136: I would remove the word "artifactitiously" (or change it).

[Response]

We removed "artifactitiously" (Line 152).

Reviewer #3

[Reviewer's comment]

This is an interesting paper where the authors present a boolean model that is able to recapitulate the WT expression of dynamics of 13 genes in the 32-cell ascidian embryo as well as predict expression in certain experimental conditions. They then do in-silico knockout experiments to conclude that their network is highly redundant, in order to then speculate that it is this redundancy in the network that underlies the evolution of early ascidian patterning via developmental systems drift.

Some clarifications are required throughout, but importantly, in order to say anything about redundancy in the network the authors should first show that the way in which they construct their networks to begin with doesn't inadvertently favour redundant networks. This is particularly important because these networks are constructed taking a bottom-up approach rather than an unbiased parameter fitting approach.

[Response]

We do not think that the regulatory functions we determined artifactually favors redundancy with the following reasons.

First, the upstream factors we used were chosen by an unbiased way. We analyzed those that are (or are expected to be) expressed at the 32-cell stage.

Second, our exhaustive search identified the simplest Boolean function in the DNF among all possible combinations. Hence, this method cannot be biased to pick redundancy.

Third, almost all redundant factors are indeed involved in regulation of target genes. For example, *Lhx3/4* is regulated by Foxd, Fgf9/16/20, and β-catenin, and we revealed that Foxd and Fgf9/16/20 give redundant cues. In embryos injected with a MO against Foxd or Fgf9/16/20, *Lhx3/4* expression is abolished (it is also abolished in β-catenin morphants). Therefore, the *Lhx3/4* function is represented as Foxd & Fgf9/16/20 & β-catenin. Then, we found that Foxd&β-catenin or Fgf9/16/20&β-catenin theoretically gave the same expression pattern as Foxd & Fgf9/16/20 & β-catenin. We do not think that this process is biased to favor redundancy. Please understand that we say that these

factors give theoretically redundant information to target genes and we never say that these factors are dispensable in real embryos.

Fourth, we picked such an example, Zfpm for *Nodal* expression, for substantiating the hypothesis. The upstream region of *Nodal* directed expression in b6.5, even after it was changed to be independent of Zfpm. This example indicates that the information given by Zfpm is indeed redundant.

To clarify these points, we added the following sentences.

> (Line 272–288) It is unlikely that the observed redundancy is an artifact derived from our method to determine RFs. First, we identified upstream regulatory factors in an unbiased way as we mentioned in the Introduction. Second, we considered all theoretically possible conjunctions to determine RFs, as we explained in detail in the Introduction; therefore, it is unlikely that this method favors or disfavors redundancy of the network. Third, RFs represent necessary and sufficient conditions for expression of individual target genes. Although more than half the regulators give theoretically redundant temporal or spatial information to target genes, they are necessary for expression of their target genes in real embryos. The above computational analysis showed that Foxd and Fgf9/16/20 theoretically gave redundant cues for regulation of *Lhx3/4*, whereas *Lhx3* expression is lost in embryos injected with an MO against *Foxd* or *Fgf9/16/20* (Tokuoka et al., 2021). Similarly, the present and previous studies have experimentally shown that each of the observed redundant factors act to establish specific expression patterns of the 13 target genes (Bertrand et al., 2003; Imai et al., 2016; Ohta and Satou, 2013; Ohta et al., 2015; Tokuhiro and Satou, 2021; Tokuhiro et al., 2017; Tokuoka et al., 2021). Exceptions include involvement of β-catenin and Gata.a in regulation of *Dmrt.a* and *Dlx.b*. Therefore, we confirmed that these factors indeed regulate expression of *Dmrt.a* and *Dlx.b* (S6 Fig). Thus, redundant temporal or spatial information is given to the 13 target genes.

[Reviewer’s comment]

Abstract

L13 (also L27 and L45): How GRNs encode dynamics and evolve are one of the most-studied and best-understood problems in biology, although I do agree that there is still a lot that we don't know. I would encourage the authors to rephrase.

[Response]

According to the advice, we revised the following sentences.

(Original) How gene regulatory networks (GRNs) encode gene expression dynamics and how GRNs evolve are not well understood.

(Revised) How gene regulatory networks (GRNs) encode gene expression dynamics and how GRNs evolve are not well understood**, although these problems have been studied extensively.** (Line 13–14)

(Original) Although regulatory relationships in gene regulatory networks have been extensively studied during the past two decades, it is not entirely clear how gene regulatory networks produce gene expression dynamics.

(Revised) Although regulatory relationships in gene regulatory networks have been studied extensively during the past two decades, it is not **fully understood** how gene regulatory networks produce gene expression dynamics. (Line 27–29)

(Original) Although the structure of such networks, which represent connections among regulatory genes, has been studied extensively, it is unclear how these networks encode gene expression dynamics.

(Revised) Although the structure of such networks, which represent connections among regulatory genes, has been studied extensively, it is **not fully understood** how these networks control gene expression dynamics. (Line 44–46)

[Reviewer's comment]

L14 (and throughout the paper): I find the terminology confusing. Why digital twin? Why not Boolean model of gene regulation?

[Response]

To avoid a possible confusion, we use “simulator” instead of “digital twin”.

[Reviewer’s comment]

L21: Playing Devil’s advocate, it might also reflect that the GRN was initially formulated to include many factors that aren’t necessary, and that might not actually be there in the embryo. How do we tease the two possibilities apart?

[Response]

We do not exclude the possibility that the reviewer pointed out, because we never say anything about the origin of such redundancy. We added a sentence in Conclusions.

> (Line 356–357) If so, it is possible that GRNs in ancient animals may have had more redundancy and that such redundancy may have decreased during evolution.

[Reviewer’s comment]

Introduction

L54-L59: More information on how RF were constructed in Tokuoka 2021 might be beneficial for the reader, particularly since the authors mention explicitly that for Nodal, this represented necessary but not sufficient conditions. The reader needs to know how these functions have been constructed, because the paper’s main claim is one of robustness of the network to evolutionary changes in the regulation. However, is these networks (RFs) are initially built assuming necessity, but with no, or little consideration, for the sufficiency, then it surely comes as no surprise that some of the components were redundant in the formulation to begin with

[Response]

This is explained in the paragraph starting with Line 76. For clarification we added several sentences to this paragraph. To explain more thoroughly how we determined RFs, we revised the following part:

(Original) …DNFs with the smallest number of conjunctions and the smallest number of upstream regulators were considered as primary candidates. These candidates were then experimentally verified. In other words, candidate DNFs were determined under the assumption that the simplest one is most likely. Then we tested whether candidate RFs correctly predicted gene expression in conditions that were not previously examined.

(Revised) …DNFs with the smallest number of conjunctions and the smallest number of upstream regulators were considered as primary candidates. In other words, candidate DNFs were determined under the assumption that the simplest one that explains all observations is most likely. In experiments for determining DNFs, we used embryos injected with morpholino antisense oligonucleotides (MO) for knockdown and/or treated with an inhibitor for the MAPK signaling pathway. Then we experimentally verified whether candidate RFs correctly predicted gene expression under conditions that were not previously examined. In verification experiments, we used MOs, synthetic mRNAs, the MAPK signaling inhibitor, and a recombinant FGF protein. In this way, we determined RFs using experiments and theoretical analyses. (Line 90–98)

We do not think that starting with finding necessary conditions artificially favors redundant RFs. Theoretical redundancy we identified in the present study cannot be found without experiments for seeking necessary conditions. The RFs we identified represent necessary and sufficient conditions for the target genes to be expressed. Therefore, it is logically unlikely that this redundancy is artifact. Please note that we never say that these theoretically redundant factors are not necessary for gene expression in ascidian embryos, but we say that these factors give redundant information to target genes.

[Reviewer’s comment]

L80: I think it is important to mention how these RFs were found in thee 2021 paper, in addition to how they are formulated. Why did the algorithms used in Tokuoka 2021 not reveal more minimal RFs, if the simplest RFs were selected?

[Response]

Our algorithms took DNFs with the smallest number of conjunctions and the smallest number of upstream regulators were considered as primary candidates among all possible DNFs. This means that 'more minimal RFs' do not explain gene expression in normal or experimental conditions. This is explained in the updated third paragraph of Introduction, which includes our answer to the preceding criticism (Line 76–100).

[Reviewer's comment]

L97 (also L55): Please clarify why the RF for Nodal is the only one out of 13 that wasn't calculated or inferred in the previous study (or did I understand this wrong?)

[Response]

We revised this part as follows:

(Original) The regulatory function (RF) for the remaining gene, *Nodal*, was also tentatively determined; it represents necessary conditions, but does not represent sufficient conditions (Tokuoka et al., 2021)

(Revised) The RF for the remaining gene, *Nodal*, has also been determined provisionally. Necessary factors for *Nodal* expression were identified by our exhaustive examination of functions of regulatory genes expressed before the 32-cell stage. However, we did not always succeed in inducing *Nodal* expression in real embryos in conditions in which the tentative RF predicted *Nodal* activation (Tokuoka et al., 2021). This suggests that an additional modulator for transcription factors or signaling pathways may be involved in *Nodal* regulation. (Line 60–66)

[Reviewer's comment]

Results

L102: Reading this sentence has made me realise that I don't quite understand how the simulations are being run. What is being simulated and how are these models initialised. From the introduction, I thought that the models are initialised using the expression at the 16-cell stage to simulate the expression at the 32-cell stage, but the sentence in L102

makes me think I have misunderstood this. Please clarify somewhere in the introduction.

[Response]

We revised the sentence as follows:

(Original) Expression patterns predicted by the tentative *Nodal* RF in various conditions are reproduced experimentally at the 32-cell stage, but not at the 16-cell stage (Tokuoka et al., 2021).

(Revised) Expression patterns predicted by the presumptive *Nodal* RF in various conditions are reproduced experimentally at the 32-cell stage (Tokuoka et al., 2021). If this *Nodal* RF represents sufficient conditions, *Nodal* expression could be induced in experimentally manipulated 16-cell embryos. However, we failed to induce *Nodal* expression in 16-cell experimental embryos, in which we reproduced conditions that were predicted to be sufficient to induce *Nodal* expression (Tokuoka et al., 2021). (Line 113–118)

[Reviewer's comment]

L104: I would have thought that if the model recapitulates the pattern at the 32-cell stage but not at the 16-cell stage, the conclusion would be that the formulation was missing something present at the 16-cell stage, not at the 32-cell stage as stated.

[Response]

There are two possibilities. If this missing factor is a repressor, it should be present at the 16-cell stage, and absent at the 32-cell stage. If this missing factor is an activator, it should be absent at the 16-cell stage, and present at the 32-ecll stage. In the former case, this factor should rapidly degrade between the 16-cell and 32-cell stages. However, this does not seem to happen. Therefore, we examined the latter possibility. For clarification, we revised the sentence as follows:

(Original) Therefore, it is likely that there is a missing factor that acts at the 32-cell stage, but not at the 16-cell stage.

(Revised) Therefore, it is likely that there is a missing **activator** that acts at the 32-cell stage, but not at the 16-cell stage. (Line 118–119)

[Reviewer's comment]

L105: I thin that when the authors say inferred here, they really mean assumed.

[Response]

We used 'hypothesized' instead of 'inferred'.

(Original) Because there is a delay between mRNA transcription and protein activity, we inferred that the mRNA encoding this missing factor should be expressed at the 16-cell stage.

(Revised) Because there is a delay between mRNA transcription and protein activity, we **hypothesized** that the mRNA encoding this missing factor should be expressed at the 16-cell stage. (Line 119–121)

[Reviewer's comment]

L133: The fourth conjunction represents experimental conditions. Which specifically?

[Response]

We revised the sentence as follows:

(Original) On the other hand, the fourth conjunction represents expression in experimental conditions, and involvement of *Zfpm* in the fourth conjunction was unclear from the above experiments.

(Revised) On the other hand, the fourth conjunction does not represent expression in normal embryos but represents expression in experimental conditions, **e.g. expression in embryos in which *Gdf1/3-r* and *Admp* are simultaneously knocked down**

(Tokuoka et al., 2021), and involvement of *Zfpm* in the fourth conjunction was unclear from the above experiments. (Line 147–150)

[Reviewer's comment]

L134-L138: I'm not sure what the perturbation is here. Is it FGF up-regulation?

[Response]

Yes. As we wrote in Line 153–154, we incubated "embryos in sea water containing FGF2, which mimicked overexpression of *Fgf9/16/20*".

[Reviewer's comment]

L153: Is the 4th conjunction an aggregate function representing every experimental perturbation then? Please clarify the difference between the 1st to 3rd conjunctions and the 4th somewhere in the introduction, and how these are inferred.

[Response]

The fourth conjunction represent expression in experimental conditions only. On the other hand, the first three conjunctions, respectively, represent expression in A6.1/A6.3/B6.1, B6.1, and b6.5 cells of normal embryos. To clarify the point, we revised the following sentence.

(Original) Next, to resolve the ambiguity included in the fourth conjunction of the previous tentative RF for Nodal, we performed additional experiments.

(Revised) There was some ambiguity in the fourth conjunction of the previous tentative RF for *Nodal*. Because the fourth conjunction does not represent expression in normal embryos, this uncertainty was not resolved in the previous study (Tokuoka et al., 2021). In the present study, to resolve this enigma, we performed additional experiments. (Line 160–163)

[Reviewer's comment]

L166-L180: Verry useful recap, thank you!

L180: Except for Foxa.a injections + FGF.

[Response]

We are sorry for the misleading sentence in the original version, but this is a misunderstanding. In embryos injected with *Foxa.a* mRNA and treated with FGF2, *Nodal* was not expressed, and this was consistent with the prediction by the RF. To clarify this point, we revised this part as follows:

(Original) As described above, if we inject *Foxa.a* mRNA into unfertilized eggs and incubate them in sea water containing FGF2 after fertilization, *Nodal* is not expressed (see S4F Fig; this condition does not satisfy any conjunctions).

(Revised) **As we have already shown in S4 Fig**, if we inject *Foxa.a* mRNA into unfertilized eggs and incubate them in sea water containing FGF2 after fertilization, *Nodal* is not expressed (this condition does not satisfy any conjunctions). (Line 188–190)

[Reviewer's comment]

L186: for wild type expression?

[Response]

We revised the sentence as follows:

(Original) On the other hand, the fourth conjunction is not necessary for expression at the 32-cell embryos.

(Revised) On the other hand, the fourth conjunction is not necessary for expression **in normal embryos** at the 32-cell stage. (Line 203–204)

[Reviewer's comment]

L187-L190: I am very confused by these statements. I am also not sure that adding a conjunction to capture experimental perturbations separately from the other 3 is rigorous. Surely in the embryo, the result of the perturbations is a consequence of the normal WT wiring of the GRN; it doesn't have a separate "perturbation/experimental" module. If the authors want to include this for the sake of the model, OK, but to then from then assume that this conjunction actually exists separately from the main, or WT GRN, and that it can evolve separately and/or be useful later in development, is a stretch.

[Response]

As we explained above, the *Nodal* RF was deduced as a Boolean formula, which explains all expression patterns in normal and experimental embryos. We do not indicate that each conjunction always corresponds to "a separate perturbation/experimental module". In addition, we showed that we were able to manipulate *Nodal* expression according to predictions based on the fourth conjunction (S4 Fig and Fig 2). To avoid possible misunderstanding, we deleted the sentences the reviews pointed out.

> (Deleted sentences) It is possible that the mechanism that the fourth conjunction represents may be evolving and may be lost in the future or may establish a new expression domain in the future. It is also possible that this conjunction may act at a later stage of the life cycle.

[Reviewer's comment]

L196: his statement is incredibly confusing. Is it the case, that the all the simulating is being done at the 16-cell stage, and that they are simulating the presence of mRNAs of different genes in different cells according to whether the proteins of these are expressed in the daughter cells at the 32-cell stage?

L199-L202 clarify the previous point. Please, try to clarify this throughout.

[Response]

The RFs describe necessary and sufficient conditions for gene expression. Therefore, if we created a condition in which a RF is satisfied, a gene with the RF should be expressed at any stage. To avoid possible misunderstanding, we revised the sentences as follows:

(Original) At the 16-cell stage, we tried to produce the pattern that is normally seen in a cell pair (b6.5) of 32-cell embryos using the digital twin, as b6.5 cells are important for patterning the neural plate at later stages (Hudson and Yasuo, 2005, 2006; Imai et al., 2006). In other words, because only *Nodal* and *Otx* are expressed in b6.5 cells of normal 32-cell embryos among the above 13 genes (Imai et al., 2004), we computationally tested various conditions to find a condition to specifically induce *Nodal* and *Otx* expression at the 16-cell stage in the digital twin. We found that *Nodal* and *Otx*, but not the other 11 genes, were expressed in the animal hemisphere of 16-cell embryos with overexpression of Sox1/2/3 and Fgf9/16/20 in the digital twin (Figure 3AB).

(Revised) **Because these RFs represent conditions necessary and sufficient for gene expression, the RFs allow us to predict expression patterns of these 13 genes even at the 16-cell stage. Therefore,** at the 16-cell stage, we tried to reproduce the pattern that is normally seen in a cell pair (b6.5) of 32-cell embryos using the RFs, as b6.5 cells are important for patterning the neural plate at later stages (Hudson and Yasuo, 2005, 2006; Imai et al., 2006). In other words, because only *Nodal* and *Otx* are expressed in b6.5 cells of normal 32-cell embryos among the above 13 genes (Imai et al., 2004), we computationally tested various conditions to find a condition to specifically induce *Nodal* and *Otx* expression at the 16-cell stage. **The simulator predicted** that *Nodal* and *Otx*, but not the other 11 genes, would be expressed in the animal hemisphere of 16-cell embryos with overexpression of Sox1/2/3 and Fgf9/16/20 (Figure 3AB) (Line 210–220)

[Reviewer’s comment]

L218: I wouldn’t call this a model prediction. The model has been constructed and selected to reproduce these patterns. Therefore, there is agreement between model and data, but this is not a model prediction.

[Response]

We are sorry that our writing was misleading. The condition we examined was not used for constructing the RFs. Therefore, we did not know before this experiment whether only *Otx* and *Nodal* were indeed expressed in the animal hemisphere at the 16-cell stage. To avoid possible misunderstanding, we revised the sentence as follows:

> (Original) In other words, the digital twin successfully reproduces gene expression dynamics of real embryos.
>
> (Revised) In other words, the simulator successfully reproduces gene expression dynamics of real embryos **even under conditions that were not used for constructing the RFs.** (Line 232–234)

[Reviewer's comment]

L222: transformed cells?

[Response]

We replaced 'transformed' with 'manipulated' (Line 237).

[Reviewer's comment]

L230: Although I agree with the results presented in the section beginning in L230, I think a more detailed discussion regarding how the original construction/inference of the networks might have favoured redundancy in the first place, is required. As I mentioned elsewhere, if the way in which these networks were constructed focused on necessity rather than sufficiency, it is possible that the algorithm had favoured networks with a high degree of redundancy to begin with. This does not mean that the GRNs in the Ascidian early embryo are redundant, in fact, they might not be, as the minimal GRN in this section shows. I don't think this is the case, and think that the authors are probably right, but a comment on whether their initial model construction favoured, or at least did not discard, redundancy the networks is needed in order for the results presented in this section to be more rigorous.

[Response]

We added a paragraph to explain the point the reviewer raised (please also see our answer to the main criticism; Line 272–288).

[Reviewer's comment]

L233: perturb?

[Response]

We do not think that 'perturb' is appropriate. We intend to change regulatory mechanisms here. For example, manipulating *cis*-regulatory regions will change regulatory mechanisms. This will lead to changes of RFs. In other words, changing RFs can simulate changes including changes in regulatory mechanisms brought by changes in *cis*-regulatory regions.

[Reviewer's comment]

L260: But you have shown above that Zfpm positively regulates Nodal (L126). Please clarify.

[Response]

We revised the sentence as follows:

(Original) To substantiate that *Nodal* expression pattern is maintained without regulation by *Zfpm* using experiments of real embryos, we tried to manipulate the upstream region of *Nodal* as below.

(Revised) That is, the simulator predicted that the *Nodal* expression pattern can be maintained without Zfpm, if the *Nodal cis*-regulatory region is properly re-designed. To substantiate this prediction, we tried to manipulate the upstream region of *Nodal*. (Line 298-300)

[Reviewer’s comment]

L256: I wonder whether the the flow of the paper might be improved if this section was to be moved to follow the section on the regulatory function of nodal?

[Response]

We thank the advice, but we do not agree with the proposed idea. This section, in which we changed the *cis*-regulatory region of *Nodal*, describes an experiment to substantiate the prediction that Zfpm gives a redundant cue for *Nodal* expression, and this prediction was described in the preceding section. Therefore, we do not think that the section in which we changed the *cis*-regulatory region of *Nodal* can be moved before the prediction appears.

[Reviewer’s comment]

Conclusions

L298: I think that here the authors are over-claiming slightly: Having all the data does not reveal the underlying the regulatory structure, hence the motivation for the modelling. I would rephrase to reflect this.

[Response]

According to the advice, we weakened the tone of the sentence the reviewer pointed out.

(Original) Therefore, we expect that we succeeded in revealing the whole regulatory system of the regulatory genes that initiate expression at the 32-cell stage.

(Revised) Therefore, we expect that we succeeded in **mathematically representing** the whole regulatory system of regulatory genes that initiate expression at the 32-cell stage. (Line 336–337)

[Reviewer's comment]

L306: The conclusion needs an in-depth exploration of whether the inferred networks might have had landed on more redundant formulations by default, or by design of the algorithms used to infer them. This is my biggest criticism of this paper: that the main claim – namely that redundancy within the GRN might have led to developmental systems drift in ascidians – is due to the networks in the early embryos of one extant species of ascidian being very redundant, where these networks have been inferred by constructing Boolean models that reproduce the data from the bottom up (not using unbiased parameter fitting or reverse-engineering for example).

[Response]

We showed that many regulatory factors give redundant cues to target genes. On the other hand, we never say that these regulatory factors, which give redundant cues, are unnecessary in real embryos. Nevertheless, they are indeed required for expression of the target genes, which we showed by experiments. What we showed in the present study are that specific expression patterns of these target genes can theoretically be achieved with smaller sets of regulatory factors than the sets used currently; information given by the upstream factors are redundant. As we explained above, for example, *Lhx3/4* expression requires Foxd, Fgf9/16/20, and β-catenin in real embryos, while the *Lhx3/4* expression pattern can be created with a combination of Foxd and β-catenin or a combination of Fgf9/16/20 and β-catenin, theoretically. This means that Foxd and Fgf9/16/20 give redundant information to *Lhx3/4* regulation. To make *Lhx3/4* independent from Foxd, loss of Foxd sites in the upstream region of *Lhx3/4* will be insufficient; probably more binding sites for Ets (an effector transcription factor activated by Fgf9/16/20 signaling) and/or for Tcf7 (β-catenin is a co-factor of Tcf7) will be required, for example (please note that this last point is beyond the scope of the present study). Namely, the regulatory mechanisms of the 13 genes contain potential redundancy, but to eliminate these potential redundancy, re-design of cis-regulatory regions are required. We believe that the above revision clarified this point.
