# Supplementary material for: A digital twin reproducing gene regulatory network dynamics of early Ciona embryos indicates robust buffers in the network
Source: PLoS Genet. 2023 Sep 27;19(9):e1010953. doi: 10.1371/journal.pgen.1010953 (PMC10530022; doi:10.1371/journal.pgen.1010953)
Supplement: Attachment — Submitted filename: ResponseRev2.pdf [file pgen.1010953.s010.pdf]

[Editors' comment]

We suggest that you give particular attention to the following two comments:

1: "In any case, for Lhx3/4, Neurogenin, and Dickkopf, it would be interesting to check if the function (Foxd & β-catenin)v(Fgf9/16/20 & β-catenin) was discarded because of the choice decided by the authors, or because it didn't comply with some experiments."

[Response]

Because expression of Lhx3/4, Neurogenin, and Dickkopf was lost in Foxd morphant embryos and in Fgf9/16/20 morphant embryos (Tokuoka et al., 2021), "Foxd&β-catenin or Fgf9/16/20&β-catenin" are incompatible. (If this function was correct, Lhx3/4, Neurogenin, and Dickkopf should be expressed in Foxd morphant embryos and in Fgf9/16/20 morphant embryos).

In addition, "Foxd&β-catenin or Fgf9/16/20&β-catenin" (2 clauses and 4 literals) is more complex than "Foxd&Fgf9/16/20&β-catenin" (1 clause and 3 literals). Therefore, even if the experiments were ignored, "Foxd&β-catenin or Fgf9/16/20&β-catenin" is not the simplest one in our definition, and cannot be the primary candidate.

We thank the advice, but we think that this topic is not within the scope of the present study but was already examined in our previous study (Tokuoka et al., 2021). To make the procedure we took clearer, we revised the following sentence.

(original) For this reason, in our previous study (Tokuoka et al., 2021), among DNFs that are compatible with gene expression patterns in normal embryos and a limited number of experimental embryos (i.e. partially filled truth tables), DNFs with the smallest number of conjunctions and the smallest number of upstream regulators were considered as primary candidates.

(revised; line 90–95) For this reason, in our previous study (Tokuoka et al., 2021), among DNFs that are compatible with gene expression patterns in normal embryos and a limited number of experimental embryos (i.e. partially filled truth tables), DNFs with the smallest number of conjunctive clauses and the smallest number of literals (upstream regulators) were considered as primary candidates; if multiple candidates were obtained, we repeated experiments until we obtained a unique candidate DNF.

[Editors' comment]

2: " how can we ensure, if a DNF with n literals is choosen, that we are not missing an additional regulator? e.g. if (AvB) as well as (AvBv¬C) were compatible with the experiments disclosing expression of X, it could be the case that in the future, a new experiment shows that indeed absence of C is required for the expression of X."

[Response]

We cannot completely rule out such a possibility. However, since we have comprehensively identified genes encoding transcription factors and signaling molecules and have examined their expression patterns (Imai et al., 2004; Tokuoka et al., 2021) (Line 126 and Line 346), it is not very likely that unknown repressor regulates the 13 genes at the 32-cell stage.

This reviewer's comment was raised as a reason for the opinion that "the redundancy analysis on this sole function is therefore not convincing". However, suppose that B in "AvB" was identified as a theoretically redundant factor in the reviewer's example. Even if it was later turned out that the real function is "AvBv¬C" but not "AvB", the previously identified redundant factor should remain to be "redundant". This is because expression patterns in all cells in normal embryos can be explained with "A" alone. This also means that "C" will be identified as a redundant factor. On the other hand, it is possible that 'A' may be newly identified as a redundant factor if '¬C' shows the identical pattern as 'A'. In any cases, the conclusion that B is a redundant factor will not be changed.

Therefore, we think that the overall conclusion (over half the regulators in regulatory functions are potentially redundant for specifying temporal and spatial gene expression patterns; Line 38-40) will not greatly be changed even if additional unknown regulator is involved (as described above, we do not think that this is very likely).

We revised the following sentence:

(original) Therefore, we expect that we succeeded in mathematically representing the whole regulatory system of regulatory genes that initiate expression at the 32-cell stage.

(revised; line 352–354) Therefore, we expect that we succeeded in mathematically representing the whole regulatory system of regulatory genes that initiate expression at the 32-cell stage, although there is a small possibility that additional hypothetical redundant factors are found in future.

[Editors' comment]

Additionally, we encourage you to replace "conjunctions" with "conjunctive clauses" when applicable, to provide the model (the set of functions) in the form of an excel sheet or, better, an SBML file and to revise the title of your study, possibly following Reviewer 3's suggestion to include "digital twin".

[Response]

We changed most of “conjunctions” to “conjunctive clauses”.

We provide the regulatory functions as an excel sheet (S2 Table).

We changed the title, which now includes “digital twin”.

Reviewer #3

[Reviewer's comment]

Reviewer #3: I thank the authors for addressing my main concern, which was that their networks might be biased towards redundancy by construction and am happy with their response and amendments to the text. I also thank their responses to my other comments. Since writing my review, it has come to my attention that the notion of a "digital twin" is gaining traction. The authors should use it instead of simulator if they wish to. I am now supportive of the publication of this paper.

[Response]

We thank the support by this reviewer. Thanks to this comment and to solve the problem raised by Reviewer 4, we reverted "simulator" to "digital twin".

(Original title) A simulator reproducing the gene regulatory network of early *Ciona* embryos indicates robust buffers in the network

(Revised title) A digital twin reproducing gene regulatory network dynamics of early *Ciona* embryos indicates robust buffers in the network

We also changed "simulator" in the text to "digital twin".

[Reviewer's comment]

As an aside, I emphatically agree with the authors that the fact that the model is Boolean, does not make it "a mere re-statement of experimental results". Boolean models have a lot of explanatory and predictive power, often more so than continuous models, and are perfectly suitable for this system where expression is on/off as opposed to graded.

[Response]

Thank you for the support. We think that our manuscript proves that our models have a predictive power.

[Reviewer's comment]

Ps. The amendment in L356-357 should read "in ancestral animals".

[Response]

We changed "ancient" to "ancestral" (line 373).

Reviewer #4

[Reviewer's comment]

IReviewer #4: This manuscript presents a program enabling the determination of expression patterns of 13 genes in 32-cell ascidian embryos from that of 18 upstream factors, which start to be expressed in the 16-cell embryo. Regulatory mechanisms are represented as Boolean functions determined in a previous work [Tokuoka et al., 2021] for 12 of these genes. The case of Nodal is revisited as the function determined in [Tokuoka et al., 2021] could not account for Nodal expression in specific situations.

Contributions of this work are the implementation of the computational tool (called simulator), a regulatory function of Nodal, and an in silico experiment to supposedly show that some regulators are redundant. Overall, the paper is interesting, but in my opinion the model definition is not convincing.

- Concerning the implemented program, it is indeed well designed with its HTML version, and might be useful for the community. However, it is a bit excessive to call it a "simulator", as it merely performs the evaluation of Boolean functions (i.e. one step, from an input pattern to a resulting pattern).

[Response]

We changed the word "simulator" to "digital twin".

[Reviewer's comment]

- My main concern relates to the RFs definition and the take home message about a supposed redundancy of many regulatory genes. The authors recognize that while "although more than half the regulators give theoretically redundant temporal or spatial information to target genes, they are necessary for expression of their target genes in real embryos." This statement sounds a bit odd to me, as I would understand "redundancy" differently.

[Response]

We add the following sentence to explain our usage of the word “redundancy”.

(added sentence; line 271–275) Note that the word “redundancy” here does not mean that redundant factors are not necessary for gene expression. Instead, it means that gene expression will not be changed without a redundant factor if regulatory regions are properly re-designed. In other words, information sufficient for specific gene expression is given to a target gene without a redundant factor.

[Reviewer’s comment]

But I am more concerned about the following claim "It is unlikely that the observed redundancy is an artifact derived from our method to determine RFs."

The problem to determine the regulatory functions is largely underdetermined as there are 2^18 (=262,144) potential truth tables for each target gene. As rightly put by the authors, it is impossible to perform so many experiments to determine the right function. When they say "we considered all theoretically possible conjunctions to determine RFs", it might be the case (note that the term "conjunctions" here is not appropriate), but they finally choose a single one, and the redundancy analysis on this sole function is therefore not convincing.

[Response]

To obtain regulatory functions of genes that begin to be expressed at the 32-cell stage, we first excluded conjunctive clauses incompatible with experimental results, and we chose the simplest one as a candidate among the remaining ones. When multiple candidates were obtained, we added experiments, and repeated the same process until we obtained a single candidate (please see our response to the next comment for the definition of “simplest”). Then, we validated obtained candidates using 16-cell embryos. In several experimental conditions, we confirmed that gene expression began precociously in 16-cell stage in the patterns that these candidates predicted. In other words, our method includes not only to choose candidates theoretically but also to validate them experimentally. This is explained in Introduction [line 99 to 100; Then we experimentally verified whether candidate RFs correctly predicted gene expression

under conditions that were not previously examined]. Because we used these verified functions, we believe that our analysis is convincing. In addition, we experimentally demonstrated that this “redundancy” is the case using *Nodal*.

To make these points clearer, we revised the following sentences:

(original) …DNFs with the smallest number of conjunctive clauses and the smallest number of literals (upstream regulators) were considered as primary candidates.

(revised; line 93–95) …DNFs with the smallest number of conjunctive clauses and the smallest number of literals (upstream regulators) were considered as primary candidates; if multiple candidates were obtained, we repeated experiments until we obtained a unique candidate DNF.

(original) Second, we considered all theoretically possible conjunctive clauses to determine RFs, as we explained in detail in the Introduction section

(revised; line 286–288) Second, we started with considering all theoretically possible conjunctive clauses to determine RFs, as we explained in detail in the Introduction section

(original) Third, RFs represent necessary and sufficient conditions for expression of individual target genes.

(revised; line 289–291) Third, RFs represent necessary and sufficient conditions for expression of individual target genes, and their sufficiency was experimentally verified (Tokuoka et al., 2021) (see also Figure 2).

(original) That is, we substantiated the prediction that rewiring to make *Nodal* independent of Zfpm does not alter the *Nodal* expression pattern.

(revised; line 344–346) That is, we substantiated the prediction that rewiring to make *Nodal* independent of Zfpm does not alter the *Nodal* expression pattern, and

demonstrated that the observed redundancy of Zfpm for *Nodal* regulation is not an artifact.

[Reviewer's comment]

First, criteria for this choice are unclear: "DNFs with the smallest number of conjunctions and the smallest number of upstream regulators were considered as primary candidates." I suppose that the authors by "conjunctions" mean "conjunctive clauses", which should be corrected. Otherwise, it would be unclear which function would be choosen between A&B&C (A and B and C) and (A&B) v (B&C) ((A and B)or(B and C), as they have the same number of literals (A,B,C) and the same number of conjunctions. The notion of simplest function here is debatable, one could say that the simplest function is the least stringent (higher number of disjunctions, i.e. clauses), as AvB is true for 3 value configurations of A and B, whereas A&B is true for only one.

[Response]

We changed "conjunctions" to "conjunctive clauses".

The definition of "simplest functions" is given in line 90–94. In the example the reviewer raised [(A and B and C) VS (A and B)or(B and C)], the former contains one conjunctive clauses and three regulators in total, while the latter contains two conjunctive clauses and four regulators in total. The number of conjunctive clauses is smaller in the former than in the latter, and the number of upstream regulators is also smaller in the former than in the latter. Therefore, in this example, the former is the primary candidate in our definition.

To make this point clearer, we revised the following sentence:

(original) …DNFs with the smallest number of conjunctions and the smallest number of upstream regulators were considered as primary candidates. In other words, candidate DNFs were determined under the assumption that the simplest one that explains all observations is most likely.

(revised; line 93–98) …DNFs with the smallest number of conjunctive clauses and the smallest number of literals (upstream regulators) were considered as primary candidates; if multiple candidates were obtained, we repeated experiments until we obtained a unique candidate DNF. In other words, candidate DNFs were determined under the assumption that the simplest DNF (DNF with the smallest number of conjunctive clauses and the smallest number of upstream regulators) that explains all observations is most likely.

[Reviewer's comment]

In any case, for Lhx3/4, Neurogenin, and Dickkopf, it would be interesting to check if the function (Foxd & β-catenin)v(Fgf9/16/20 & β-catenin) was discarded because of the choice decided by the authors, or beacuse it didn't comply with some experiments.

[Response]

Because expression of *Lhx3/4*, *Neurogenin*, and *Dickkopf* was lost in *Foxd* morphant embryos and in *Fgf9/16/20* morphant embryos (Tokuoka et al., 2021), "Foxd&β-catenin or Fgf9/16/20&β-catenin" are incompatible. (If this function was correct, *Lhx3/4*, *Neurogenin*, and *Dickkopf* should be expressed in *Foxd* morphant embryos and in *Fgf9/16/20* morphant embryos).

In addition, "Foxd&β-catenin or Fgf9/16/20&β-catenin" (2 clauses and 4 literals) is more complex than "Foxd&Fgf9/16/20&β-catenin" (1 clause and 3 literals). Therefore, even if we ignore the experimental results, "Foxd&β-catenin or Fgf9/16/20&β-catenin" is not the simplest one and cannot be the primary candidate.

We thank the advice, but we think that this topic is not within the scope of the present study but was already examined in our previous study (Tokuoka et al., 2021). To make the procedure we took clearer, we revised the following sentence.

(original) For this reason, in our previous study (Tokuoka et al., 2021), among DNFs that are compatible with gene expression patterns in normal embryos and a limited number of experimental embryos (i.e. partially filled truth tables), DNFs with the smallest number of conjunctions and the smallest number of upstream regulators were considered as primary candidates.

(revised; line 90–95) For this reason, in our previous study (Tokuoka et al., 2021), among DNFs that are compatible with gene expression patterns in normal embryos and a limited number of experimental embryos (i.e. partially filled truth tables), DNFs with the smallest number of conjunctive clauses and the smallest number of literals (upstream regulators) were considered as primary candidates; if multiple candidates were obtained, we repeated experiments until we obtained a unique candidate DNF.

[Reviewer's comment]

Second, how can we ensure, if a DNF with n literals is choosen, that we are not missing an additional regulator? e.g. if (AvB) as well as (AvBv¬C) were compatible with the experiments disclosing expression of X, it could be the case that in the future, a new experiment shows that indeed absence of C is required for the expression of X.

[Response]

We cannot completely rule out such a possibility. However, since we have comprehensively identified genes encoding transcription factors and signaling molecules and have examined their expression patterns (Imai et al., 2004; Tokuoka et al., 2021) (Line 127 and Line 349), it is not very likely that unknown repressor regulates the 13 genes at the 32-cell stage.

This reviewer's comment was raised as a reason for the opinion that "the redundancy analysis on this sole function is therefore not convincing". However, suppose that B in "AvB" was identified as a theoretically redundant factor in the reviewer's example. Even if it was turned out that the real function is "AvBv¬C" but not "AvB", the previously identified redundant factor should remain to be "redundant". This is because expression patterns in all cells in normal embryos can be explained only with "A". This also means that "C" is also a redundant factor. On the other hand, it is possible that 'A' may be newly identified as a redundant factor. In any cases, the conclusion that B is a redundant factor will not be changed.

Therefore, we think that the overall conclusion (over half the regulators in regulatory functions are potentially redundant for specifying temporal and spatial gene expression patterns; Line 38-39) will not greatly be changed even if additional unknown regulator is involved (as described above, we do not think that this is very likely).

[Reviewer's comment]

In Nodal's function, I am surprised to see that β-catenin appears as an activator and an inhibitor. Such cases are not so common. It would be good to check again an explain why β-catenin acts as a dual regulator.

[Response]

This issue is resolved by a previous study (Oda-Ishii et al., 2016). This reference is cited in the main text, and the mechanism is briefly explained in the legend for Figure 4 (Similarly, because β-catenin antagonizes Gata.a activity, the term "¬β-catenin" implies that Gata.a acts).

We thank the comment, but we do not think that we need to explain thoroughly how β-catenin acts, because it will not be essential for understanding the present paper. Therefore, we do not revise the manuscript in response to this comment.

[Reviewer's comment]

I suppose that there exist data on binding sites in ascidian, and it would be interesting to use these data to help choosing the RFs (choice which is so far based on expression data).

[Response]

Thank you for an interesting suggestion. This method will be possible for genes, of which upstream regulatory regions are dissected extensively. On the other hand, to our knowledge, *in silico* identification of biding sites do not necessarily ensure that identified sites work, and upstream regions of these 13 genes have not been analyzed thoroughly by experiments. Therefore, we think that this analysis is beyond the scope of the present study.

[Reviewer's comment]

In Table S1, it appears clearly that Foxd, Fgf9/16/20 and β-catenin do not have the same patterns of expression in the 16-cell and the 32-cell embryo. This would imply that they are regulated, but I am not sure the authors provide any comment on this point, neither on potential cell-cell signaling. I also wonder if there are some cross regulations, or circuits, as the proposed network is free of loops.

[Response]

As we explained in line 107, distribution of upstream factors is based on observations in previous studies, and we assumed that descendants of cells expressing a transcription factor gene at the 16-cell stage express the encoded protein at the 32-cell stage because of a delay between gene expression and protein translation.

(1) Foxd transcription factor protein is expected to be present in daughter cells of cells that expressed Foxd gene at the 16-cell stage. At the 16-cell stage, Foxd protein is not expected anywhere, because Foxd gene is not activated before the 16-cell stage.
(2) Cells that receive Fgf9/16/20 signaling molecule were determined by antibody staining for dpERK in normal and experimental conditions in previous studies (Hudson et al., 2003; Ohta and Satou, 2013; Tokuoka et al., 2018).
(3) Cells with nuclear β-catenin were determined by antibody staining for β-catenin, which was done in a previous study (Hudson et al., 2013).

Our manuscript addresses genes that begins to be expressed at the 32-cell stage, and therefore regulation of these upstream factors is not within the scope of the present study.

It is possible that genes encoding the upstream factors are regulated at later stages by the factors encoded by the genes that begin to be expressed at the 32-cell stage, and these factors may eventually constitute regulatory loops. However, it is not within the scope of the present study.

Cross-regulations are also possible, but it is very unlikely that genes that begin to be expressed at the 32-cell stage regulate one another at the 32-cell stage, because of a delay between gene expression and protein translation.

These possible regulations are not included in the RFs we used in the present study, as we discussed in Line 354–357 [On the other hand, many regulatory genes analyzed in the present study are also expressed in later stages of the life cycle (Imai et al., 2004).

Such expression will be regulated by different mechanisms, and these mechanisms may not be included in the RFs we used in the present study.]

[Reviewer's comment]

Finally, Boolean functions to model regulatory networks driving development have been used for several decades now. There are many studies, for example L. Sánchez & D. Thieffry as well as R. Albert & HG Othmer modelling work on the regulatory modules controlling drosophila segmentation, ER Álvarez-Buylla papers on Arabidopsis development.

[Response]

We added the suggested references in Line 78.

[Reviewer's comment]

There are also software tools devoted to Boolean models (GINsim, BoolNet,...).

I would advise to provide the model (the set of functions) in the form of an excel sheet or, better, as an SBML file.

[Response]

We provide regulatory functions in an excel sheet (Table S2).

[Reviewer's comment]

Overall, the manuscript would deserve a carefull re-reading as there are some ill-constructed sentences.

The title should be changed. A simulator does not reproduce a network, rather its dynamics. Moreover, as previously mentionned the term "simulator" does not seem appropriate here.

[Response]

We carefully checked the manuscript again. We are sorry for unreadable sentences.

The title was changed as follows:

(Original title) A simulator reproducing the gene regulatory network of early *Ciona* embryos indicates robust buffers in the network

(Revised title) A digital twin reproducing gene regulatory network dynamics of early *Ciona* embryos indicates robust buffers in the network
